# Mitigation of aflatoxin contamination of maize, groundnut, and sorghum by commercial biocontrol products in farmers’ fields across Burkina Faso, Mali, Niger, and Togo

**DOI:** 10.1186/s43170-024-00313-3

**Published:** 2024-11-11

**Authors:** Saïdou Bonkoungou, Karim Dagno, Adamou Basso, Tedihou Ekanao, Joseph Atehnkeng, Daniel Agbetiameh, Adama Neya, Mahama Toure, Assiata Tiendrebeogo, Mamadou Konate, Bibata Outani, Matieyedou Konlambigue, Kenneth A. Callicott, Peter J. Cotty, Ibnou Dieng, Titilayo D. O. Falade, Ranajit Bandyopadhyay, Alejandro Ortega-Beltran

**Affiliations:** 1 Institut de l’Environnement et de Recherches Agricoles, Ouagadougou, Burkina Faso; 2 Institut d’Économie Rurale, Bamako, Mali; 3 Institut National de la Recherche Agronomique du Niger, Niamey, Niger; 4 Institut Togolais de Recherche Agronomique, Lomé, Togo; 5 International Institute of Tropical Agriculture (IITA), Ibadan, Nigeria; 6 IITA, Kigali, Rwanda; 7 United States Department of Agriculture, Agricultural Research Service, Tucson, AZ USA; 8 College of Food Science and Engineering, Ocean University of China, Qingdao, China; 9 Present Address: IITA, Bukavu, Democratic Republic of Congo; 10 Present Address: Department of Crop and Soil Sciences, Kwame Nkrumah University of Science and Technology, Kumasi, Ghana

**Keywords:** Aflatoxin biocontrol, West Africa, Long-term efforts, Smallholder agriculture

## Abstract

**Background:**

Aflatoxin contamination by *Aspergillus* section Flavi fungi poses a significant threat to food security and public health in sub-Saharan Africa (SSA). Maize, groundnut, and sorghum are staple crops frequently contaminated with aflatoxins, sometimes at dangerous levels. Despite its detrimental effects, many farmers in SSA lack access to effective tools for mitigating aflatoxin contamination. Biocontrol based on atoxigenic isolates of *A. flavus* is an effective tool to limit aflatoxin contamination.

**Methods:**

The development, testing, registration, and commercial use of the aflatoxin biocontrol product Aflasafe BF01 for use in Burkina Faso is described. In addition, the deployment of the biocontrol technology across Mali, Niger, and Togo is documented, and for the first time, the use of aflatoxin biocontrol in sorghum is reported.

**Results:**

In all four countries, treated crops had significantly (*P* < 0.05) less aflatoxins than crops from untreated fields. Most treated crops met the stringent tolerance threshold for human consumption, 4 ppb total aflatoxin. Using native atoxigenic isolates of *A. flavus* and employing a multi-disciplinary approach, aflatoxin biocontrol products have demonstrated significant success in reducing aflatoxin levels in treated crops compared to untreated ones.

**Conclusions:**

This multi-year, multi-funded source study underscores the effectiveness of biocontrol strategies in mitigating aflatoxin contamination at scale, offering a regional approach for sustainable management in West Africa and potentially unlocking significant health and economic benefits for the region.

**Supplementary Information:**

The online version contains supplementary material available at 10.1186/s43170-024-00313-3.

## Introduction

In sub-Saharan Africa (SSA), maize, groundnut, and sorghum are important staple crops. However, these crops are frequently contaminated with the highly toxic and carcinogenic aflatoxins by *Aspergillus* section Flavi fungi (Bandyopadhyay et al. 2007; Frisvad et al. 2019; Udomkun et al. 2017). The contamination negatively affects health, income, productivity, and trade sectors (Logrieco et al. 2018; Matumba et al. 2017; Xiong and Beghin 2012). Unfortunately, most farmers in SSA who grow susceptible crops often lack knowledge of and access to effective tools to protect their crops from aflatoxin, leading to high levels of aflatoxin exposure in the region (Baglo et al. 2020; Falade et al. 2022; Jelliffe et al. 2023; Johnson et al. 2018; Waliyar et al. 2015; Warth et al. 2012).

The major causal agent of contamination across the globe, regardless of cropping systems, is *A. flavus* (Amaike and Keller 2011). This fungus is composed of two morphotypes, the L and S, which differ in genetic, physiological, and morphological characteristics (Cotty 1989; Ohkura et al. 2018; Singh et al. 2020). Both morphotypes produce B aflatoxins. However, S morphotype genotypes consistently produce high aflatoxin levels while L morphotype fungi produce variable levels from no aflatoxins up to levels comparable to those by S morphotype fungi (Cotty 1997). In West Africa, fungi resembling the S morphotype but producing both B and G aflatoxins are associated with various crops and for decades were known as unnamed taxon S_BG_ (Atehnkeng et al. 2008a; Cotty and Cardwell 1999; Diedhiou et al. 2011; Probst et al. 2014). However, in-depth molecular studies have revealed that the unnamed taxon S_BG_ fungi may belong to *A. cerealis*, *A. aflatoxiformans*, *A. austwickii*, *A. minisclerotigenes*, or taxa yet to be named (Frisvad et al. 2019; Singh and Cotty 2019). In some studies lacking molecular characterization, fungi resembling the S morphotype of *A. flavus* have been referred to as ‘fungi with S morphology’ (Atehnkeng et al. 2022; Senghor et al. 2021).

Aflatoxin contamination can occur both pre- and post-harvest (Diao et al. 2014; Hell et al. 2008; Mahuku et al. 2019). Thus, effective management strategies must be designed using crop value chain approaches converging technological, policy, and institutional options (Ortega-Beltran and Bandyopadhyay 2023). A technological option is the use of biocontrol products containing atoxigenic genotypes of *A. flavus* as active ingredients. The term “atoxigenic” refers to organisms that do not produce aflatoxins although they may produce other metabolites, including cyclopiazonic acid (CPA), Aspergillic acid, and/or Kojic acid (Ortega-Beltran and Bandyopadhyay 2023). Application of atoxigenic genotypes alters the composition of *A. flavus* populations so that atoxigenic *A. flavus* fungi are more common and aflatoxin-producers are greatly reduced. These changes to fungal populations extend beyond treated fields and persist over several years, providing area-wide and multi-season benefits.

Use of atoxigenic genotypes of *A. flavus* as biocontrol agents for the prevention of aflatoxin contamination was developed and registered by the United States Department of Agriculture—Agricultural Research Service (USDA-ARS) for use on cotton. Registered uses were subsequently expanded to include maize, groundnut, pistachio, almond, and fig grown in the US (Cotty et al. 2007; Dorner 2004; Doster et al. 2014; Ortega-Beltran et al. 2019). The International Institute of Tropical Agriculture (IITA), USDA-ARS, and several national and international institutions adapted and improved the technology for use in SSA under the tradename Aflasafe (Bandyopadhyay et al. 2016). These biocontrol products allow smallholder farmers to grow susceptible crops in aflatoxin-prone areas across SSA while meeting strict regulatory thresholds of domestic and foreign premium markets (Bandyopadhyay et al. 2019; Mahuku et al. 2022; Senghor et al. 2021). Moreover, lower aflatoxin levels are obtained across the value chain if biocontrol is part of an integrated management strategy. However, developing, testing, registering, and manufacturing and distributing an aflatoxin biocontrol product commercially available for large-scale use requires a long-term, multi-disciplinary approach (Ortega-Beltran and Bandyopadhyay 2023).

IITA and partners initially developed products for use in single countries (e.g., Aflasafe for Nigeria, Aflasafe KE01 for Kenya). Later, a product initially developed for Senegal, Aflasafe SN01 (Senghor et al. 2020), was subsequently made available for use in The Gambia once the active ingredients were shown to be endemic to both countries (Senghor et al. 2021). Thus, Aflasafe SN01 became the first aflatoxin biocontrol product commercialized for use in two countries.

Aflasafe SN01 is registered with the Comité Sahélien des Pesticides (CSP) of the Comité permanent Inter-Etats de Lutte contre la Sécheresse dans le Sahel (CILSS). A product registered with CSP/CILSS can be used in all CILSS member countries: Benin, Burkina Faso, Cape Verde, Chad, Côte d'Ivoire, The Gambia, Guinea Bissau, Mali, Mauritania, Niger, Senegal, and Togo. Expanding use across those countries can drastically cut product development, testing, registration, and scaling cost and time, for the benefit of farmers, consumers, and industries in need of aflatoxin management strategies. However, this may not be an appropriate practice for a product like Aflasafe, which works best when genotypes of the active ingredient fungi of a registered product are native to a target country (Bandyopadhyay et al. 2016; Probst et al. 2011). Because of their adaptation to local cropping systems, native fungi have better chances to reduce aflatoxin contamination compared to exotic strains (Mehl et al. 2012; Moral et al. 2020; Bandyopadhyay et al. 2022). Therefore, if the genetic groups of the active ingredients of an Aflasafe product registered with CSP/CILSS are also native to other CILSS countries then it makes sense to use that product in multiple countries, as in the case of The Gambia described above (Senghor et al. 2021).

In the current study, we report the pathway to develop the biocontrol product Aflasafe BF01, its testing in hundreds of farmers’ fields in multiple agroecologies in Burkina Faso during 2012 and 2013, its registration in 2017 with CSP/CILSS, and its use in commercial agriculture in efforts led by a private sector company. The effectiveness of Aflasafe BF01 in limiting aflatoxin in crops produced in thousands of fields of maize, groundnut, and sorghum across Mali, Niger, and Togo from 2019 to 2023 is also reported. In addition, effectiveness of Aflasafe BF01 was compared with Aflasafe SN01 in Mali. Overall, the use of the aflatoxin biocontrol technology resulted in over 80% less aflatoxin in treated crops compared to untreated crops, in all four countries. This is the first report of aflatoxin mitigation efforts at such a scale in a true regional approach for the sustainable management of aflatoxins in West Africa, which can pave the way for obtaining health and economic benefits in the region.

## Materials and methods

### Biocontrol product development to commercialization in Burkina Faso

The protocols for sample preparation and microbiological, molecular, and aflatoxin analysis used in the current study are given in brief here as they have been described in previous studies on *Aspergillus* characterization and aflatoxin biocontrol development (Agbetiameh et al. 2019; Bandyopadhyay et al. 2019; Mahuku et al. 2022; Senghor et al. 2021).

**Fig. 1:**
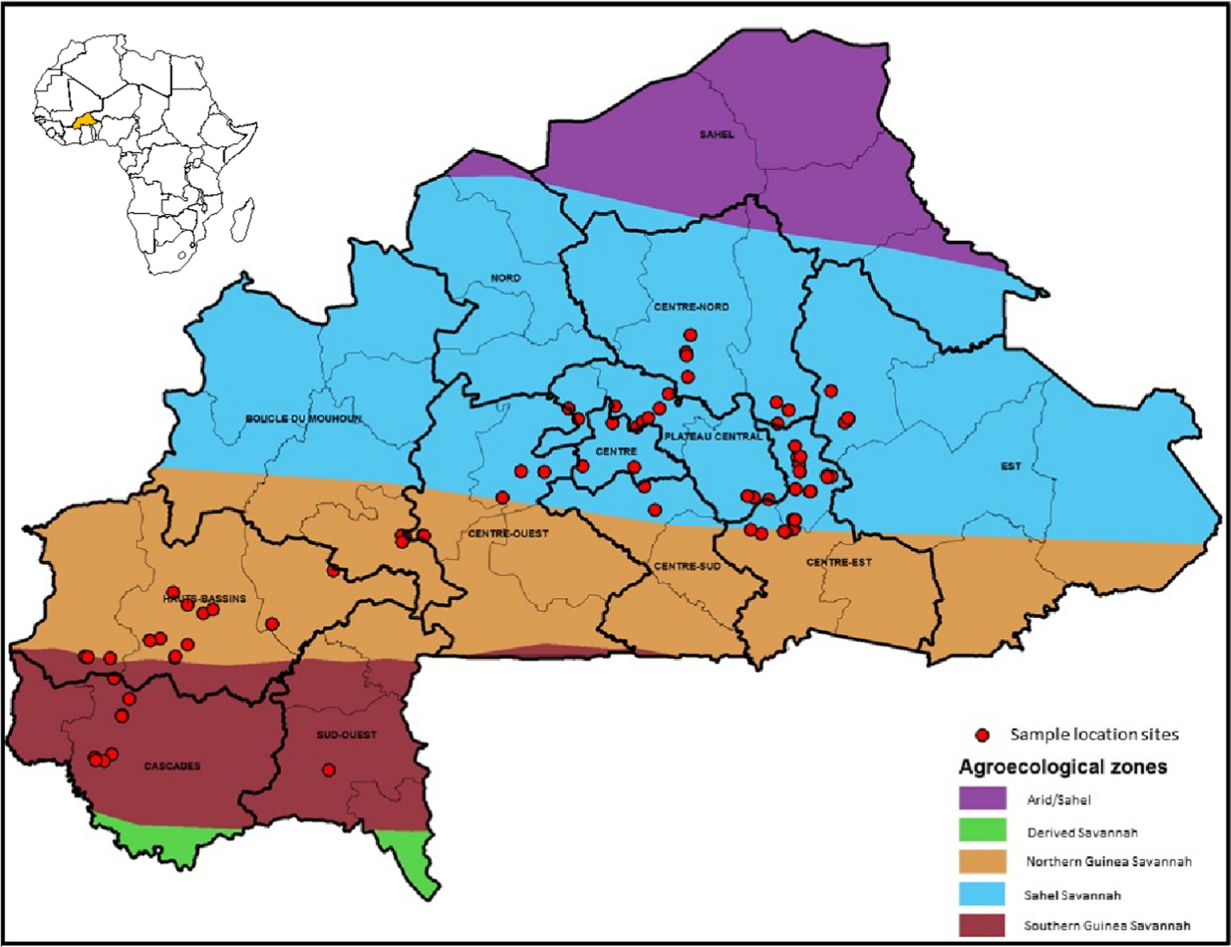
Map of Burkina Faso indicating the location of fields cropped to maize or groundnut that were sampled (both grain and soil) during 2010 to identify atoxigenic isolates of *Aspergillus flavus* L morphotype in three agroecological zones. In the African continent map (upper left), Burkina Faso is highlighted in orange

**Sample collection in Burkina Faso.** In 2010, 122 paired samples (61 grain, 61 soil) from maize fields and 104 paired samples (52 kernel, 52 soil) from groundnut fields were collected across three agroecological zones (AEZs) (Fig. 1). Provinces (16 total) and the number of samples per province are described in Suppl. Table 1. Samples were air-dried and transported to Institut de l'Environnement et de Recherches Agricoles (INERA), Plant Pathology Laboratory in Ouagadougou, Burkina Faso, prior to shipment to the Pathology and Mycotoxin Unit of IITA-Ibadan, Nigeria, under import/export permits from phytosanitary authorities in Burkina Faso and Nigeria. In Ibadan, all grain and soil samples were dried in an air-forced oven (48 h, 50 °C). Grain processing and homogenization were described in a study reporting aflatoxin prevalence in crops grown in Burkina Faso, Mali, and Niger (Falade et al. 2022). Soil processing was conducted as described in a study examining communities of aflatoxin-producing fungi from maize soils collected in Nigeria (Donner et al. 2009).

***Aspergillus* section Flavi populations and densities.** *Aspergillus* section Flavi fungi associated with the soil, maize, and groundnut were characterized. The dilution plate technique on modified Rose Bengal agar was used (Cotty 1994). Fungal densities were calculated as colony-forming units (CFU) per g of sample (Suppl. Table 1). Isolates were identified as either the *A. flavus* L morphotype, fungi with S morphology, *A. parasiticus*, or *A. tamarii* (Table 1) based on their colony characteristics and spore ornamentation (Cotty 1989; Cotty and Cardwell 1999; Klich and Pitt 1988). All isolates were saved as agar plugs (3 mm dia) of sporulating cultures in 4 ml vials containing 2 ml sterile distilled water and maintained at room temperature.

**Table 1:** Frequencies of *Aspergillus* species in maize and groundnut soil and grain samples collected in 2010 across 16 provinces in three agroecological zones (AEZ) of Burkina Faso

AEZ^a^	Province	*Aspergillus* species/morphotype distribution (%)^b^																	
		Maize									Groundnut								
			Soil				Grain					Soil				Grain			
		n^c^	L	S	P	T	L	S	P	T	n^c^	L	S	P	T	L	S	P	T
NGS	Balé	4	98	2	0	0	100	0	0	0	2	100	0	0	0	100	0	0	0
	Boulgou	5	95	4	0	1	96	0	0	4	3	100	0	0	0	100	0	0	0
	Houet	6	82	13	0	5	100	0	0	0	4	80	20	0	0	100	0	0	0
	Kénédougou	1	47	40	0	13	100	0	0	0	3	69	27	0	4	80	0	20	0
	Kouritenga	5	98	1	0	1	100	0	0	0	3	83	17	0	0	100	0	0	0
SGS	Cascades	1	73	0	0	27	100	0	0	0	1	53	47	0	0	100	0	0	0
	Comoé	5	88	10	0	2	100	0	0	0	4	65	29	0	6	100	0	0	0
SS	Bazéga	2	100	0	0	0	100	0	0	0	2	100	0	0	0	100	0	0	0
	Boulkiemdé	4	95	4	0	1	100	0	0	0	4	98	2	0	0	100	0	0	0
	Gnagna	5	100	0	0	0	100	0	0	0	5	100	0	0	0	100	0	0	0
	Gourma	7	97	3	0	0	99	0	0	1	7	100	0	0	0	98	2	0	0
	Kadiogo	4	100	0	0	0	99	0	0	1	–	–	–	–	–	–	–	–	–
	Komandjari	4	100	0	0	0	100	0	0	0	5	100	0	0	0	100	0	0	0
	Kourwéogo	1	100	0	0	0	100	0	0	0	–	–	–	–	–	–	–	–	–
	Oubritenga	5	100	0	0	0	95	5	0	0	5	100	0	0	0	100	0	0	0
	Sanmatenga	2	100	0	0	0	100	0	0	0	3	100	0	0	0	100	0	0	0

^a^NGS: Northern Guinea Savannah; SGS: Southern Guinea Savannah; SS: Sahel Savannah

^b^L: *A. flavus* L morphotype; S: fungi with S morphology; P: *A. parasiticus*; T: *A. tamarii*

^c^For each crop, grain and soil samples were obtained from the same field

**Aflatoxin-producing abilities of *A. flavus* L morphotype isolates.** A total of 2629 isolates of *A. flavus* L morphotype were recovered and evaluated for their abilities to produce aflatoxins (Table 2). Each isolate was independently inoculated on autoclaved maize kernels and incubated for 7 days at 31 °C (Probst and Cotty 2012). Aflatoxins were extracted and quantified using thin layer chromatography and scanning densitometry as previously described (Probst and Cotty 2012). Isolates without detectable levels of aflatoxins were classified as atoxigenic. The aflatoxin B1 limit of detection was 1 part per billion (ppb).

**Table 2:** Frequencies of toxigenic and atoxigenic *Aspergillus flavus* L morphotype isolates recovered from maize and groundnut soil and grain samples collected in 2010 across 16 provinces in three agroecological zones (AEZ) of Burkina Faso

AEZ^**a**^	Province	Maize soil			Maize grain			Groundnut soil			Groundnut grain		
		n	% Atox	% Tox^**b**^	n	% Atox	% Tox^**b**^	n	% Atox	% Tox^**b**^	n	% Atox	% Tox^**b**^
NGS	Balé	55	12	88 (41,450)	24	47	53 (34,205)	24	10	90 (52,370)	27	13	87 (31,160)
	Boulgou	44	15	85 (25,700)	38	44	56 (51,220)	38	25	75 (31,230)	42	23	77 (60,320)
	Houet	80	20	80 (56,420)	82	16	84 (40,800)	82	10	90 (69,880)	35	0	100 (72,800)
	Kénédougou	24	36	64 (17,890)	18	20	80 (21,470)	18	13	87 (220,100)	30	0	100 (57,330)
	Kouritenga	75	16	84 (131,670)	–	–	–	48	10	90 (65,334)	66	2	98 (23,710)
SGS	Cascades	9	40	60 (80,420)	9	0	100 (62,600)	9	7	93 (8,180)	13	0	100 (38,570)
	Comoé	69	16	84 (67,690)	50	29	71 (58,580)	50	13	87 (71,410)	44	21	79 (47,500)
SS	Bazéga	35	18	82 (78,320)	39	9	91 (45,560)	39	3	97 (43,590)	30	0	100 (72,270)
	Boulkiemdé	39	27	73 (66,640)	48	9	91 (70,300)	48	0	100 (62,980)	38	9	91 (45,800)
	Gnagna	65	11	89 (53,700)	49	10	90 (70,870)	49	8	92 (64,920)	74	0	100 (33,150)
	Gourma	94	10	90 (84,460)	57	25	75 (39,370)	57	7	93 (56,700)	75	6	94 (72,540)
	Kadiogo	55	7	93 (51,950)	43	15	85 (42,580)	–	–	–	–	–	–
	Komandjari	46	8	92 (64,660)	35	42	58 (143,200)	35	5	95 (69,310)	41	7	93 (46,610)
	Kourwéogo	10	33	67 (72,420)	82	0	100 (54,930)	–	–	–	–	–	–
	Oubritenga	58	15	85 (33,160)	57	17	83 (50,150)	57	10	90 (42,964)	56	19	81 (54,190)
	Sanmatenga	27	7	93 (59,370)	23	17	83 (58,670)	23	4	96 (73,360)	42	2	98 (45,040)
Mean			18	82		20	80		9	91		8	92

^a^NGS: Northern Guinea Savannah; SGS: Southern Guinea Savannah; SS: Sahel Savannah

^b^Aflatoxin-producing ability was determined by independently inoculating each L morphotype isolate in five grams of autoclaved maize kernels, followed by incubation for seven days at 31 °C. Aflatoxins were extracted and quantified as previously described (Probst et al. 2012). Number in parenthesis is the average aflatoxin B1 producing potential (ppb) of the toxigenic isolates recovered from each province in each substrate

**Deletions in aflatoxin and cyclopiazonic acid gene clusters.** From the 337 atoxigenic isolates of the *A. flavus* L morphotype, 148 were sent to the USDA-ARS Aflatoxin Reduction in Crops Laboratory in Tucson, Arizona under an USDA’s Animal and Plant Health Inspection Service (APHIS) Permit to Move Live Plant Pests and Noxious Weeds. Indels in 32 gene markers involved in the production of aflatoxins and cyclopiazonic acid (CPA) were monitored using a multiplex-PCR assay known as cluster amplification patterns (CAPs) (Callicott and Cotty 2015). DNA extraction and multiplex-PCR were conducted as previously described (Callicott and Cotty 2015).

**Mating-type idiomorph characterization.** The mating-type idiomorphs of the 148 atoxigenic isolates were characterized through multiplex-PCR amplification of *MAT1-1* and *MAT1-2* segments, utilizing primers M1F, M1R, M2F, and M2R as described in Ramirez-Prado et al. (2008).

**Microsatellite genotyping.** The 148 atoxigenic isolates were genotyped using 17 simple sequence repeat (SSR) markers following the method of Islam et al. (2018). Eight isolates did not amplify one marker but were successfully genotyped at the remaining 16 markers. Unamplified or uncalled alleles were treated as missing data, which represented 0.3% of the data. Minimum spanning networks (one classifying isolates by their AEZ of origin and another by their CAPs deletion pattern and mating-type) were created using Bruvo distances (Bruvo et al. 2004) within the *poppr* R package (Kamvar et al. 2014, 2015).

**Criteria for selecting atoxigenic isolates to compose the aflatoxin biocontrol product.** Deletions in aflatoxin and CPA gene clusters, membership to vegetative compatibility groups (VCGs) exclusively composed of atoxigenic members, number of locations where the atoxigenic VCG was detected, host from which originally isolated, and abilities to limit aflatoxin contamination in competition experiments were the criteria used to select four atoxigenic isolates to constitute an experimental biocontrol product (Table 3).

**Table 3:** Information on the four atoxigenic isolates of *Aspergillus flavus* selected as active ingredient for evaluation in an experimental biocontrol product under farmer field conditions

Isolate	Atoxigenic African *Aspergillus* VCG^a^	No. of atoxigenic members in the VCG	No. of locations with atoxigenic members	No. of toxigenic members	Basis for selection
G018-2	BF018	1	1	0	Large deletions in aflatoxin gene cluster, isolated from groundnut kernels, abilities to reduce aflatoxin contamination
M011-8	BF011	15	9	0	Wide distribution of atoxigenic members, isolated from maize kernels
M109-2	BF109	7	4	0	Wide distribution of atoxigenic members, isolated from maize kernels
M110-7	BF110	13	8	0	Large deletion in aflatoxin gene cluster, wide distribution of atoxigenic members, isolated from maize kernels

^a^VCG: vegetative compatibility group

**Fig. 2:**
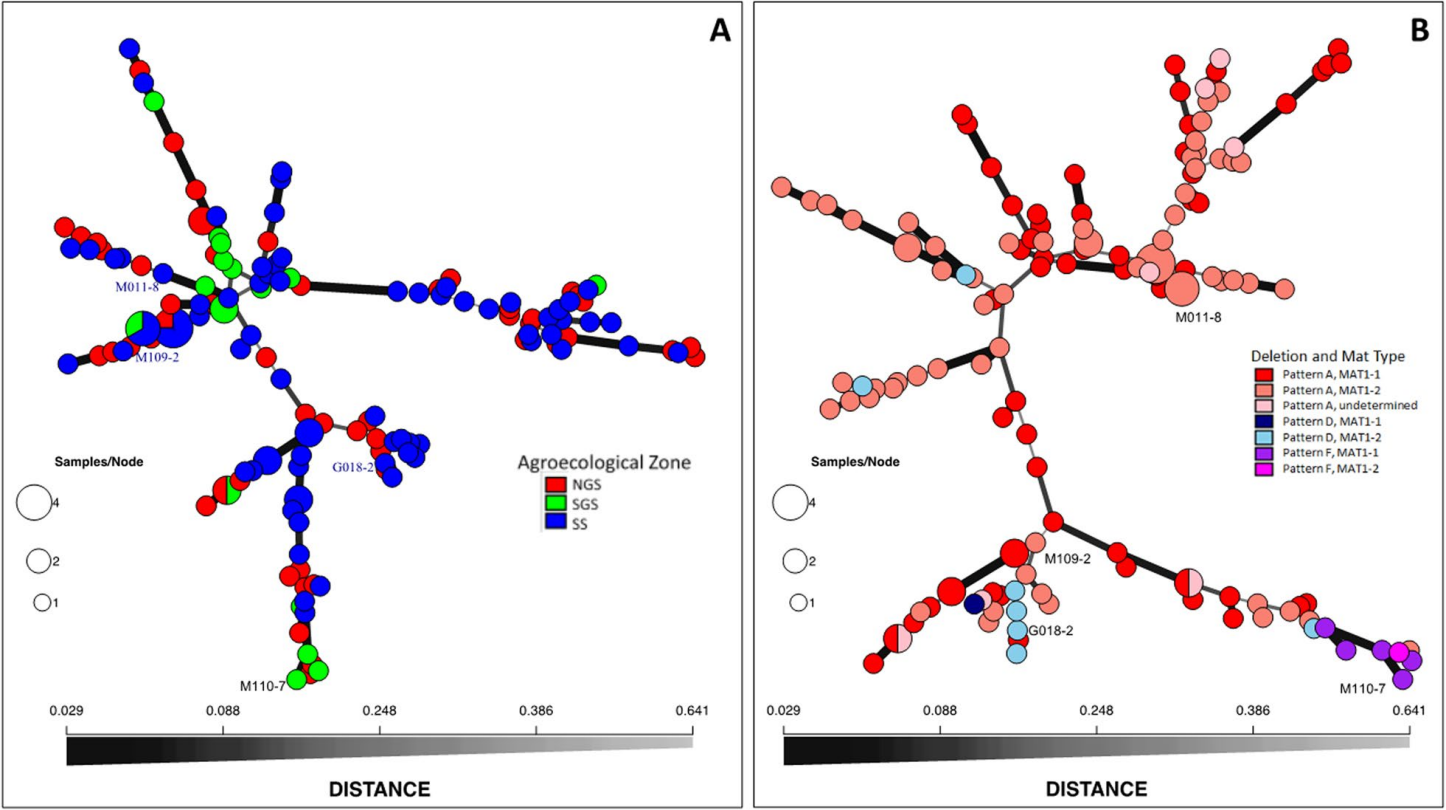
Minimum spanning networks of the 136 haplotypes found in 148 *Aspergillus flavus* isolates from Burkina Faso. The four haplotypes eventually selected to compose the aflatoxin biocontrol Aflasafe BF01 are indicated: M011-8, G018-2, M110-7, and M109-2. Genetic distances were calculated using Bruvo distances from 17 Simple Sequence Repeat (SSR) markers. The first network (**A**) classifies the isolates by their agroecological zone of origin (NGS: Northern Guinea Savannah; SGS: Southern Guinea Savannah; SS: Sahel Savannah). The second network (**B**) classifies the isolates based on their CAPs deletion pattern and mating type idiomorph

**Fig. 3:**
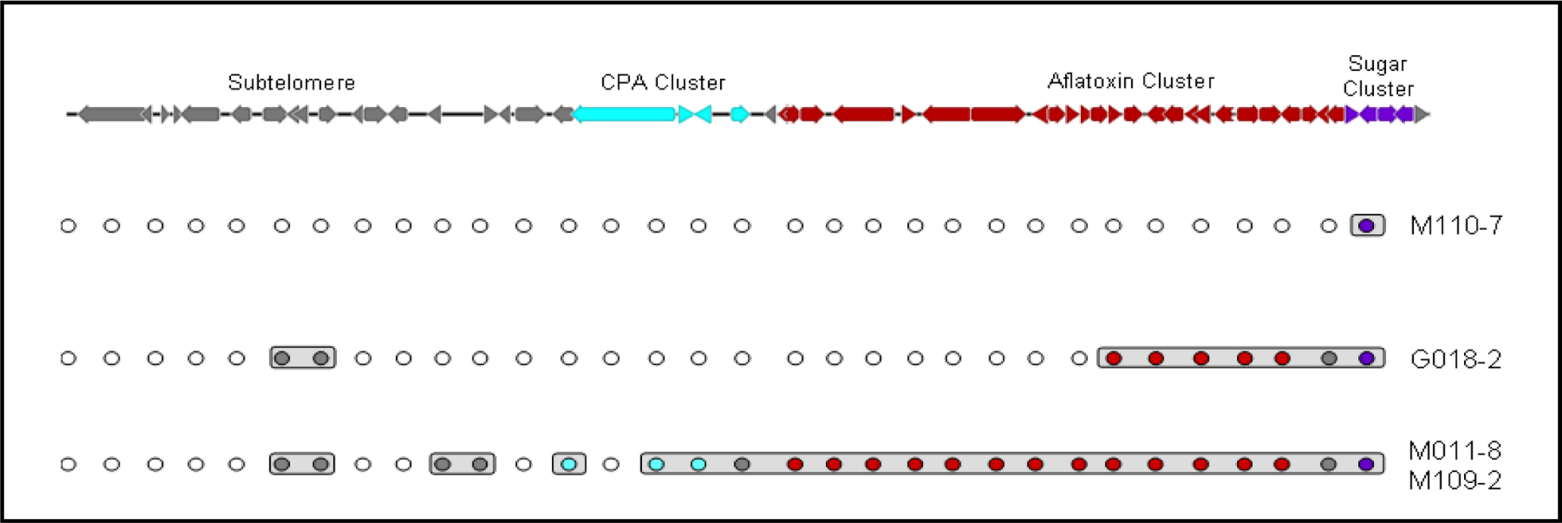
Graphical representation of PCR amplifications of the sub-telomere region of chromosome 3 containing the aflatoxin and CPA clusters for the four atoxigenic isolates from Burkina Faso that are the active ingredients of the aflatoxin biocontrol product Aflasafe BF01. Open circles = no amplification; filled circles = successful amplification

**Tester pairs of selected biocontrol isolates for vegetative compatibility analyses.** For the isolates selected to compose the experimental biocontrol product (Figs. 2, 3), tester pairs for vegetative compatibility analyses (VCA) were obtained by generating mutants and pairing complementary nitrate-non-utilizing mutants (Ortega-Beltran and Cotty 2018). A random set of 500 aflatoxin-producing isolates of the *A. flavus* L morphotype was checked for vegetative compatibility by pairing mutants generated as above with the tester pairs of the VCGs composing the experimental biocontrol product (*data not shown*).

**Manufacturing of the experimental biocontrol product.** An experimental biocontrol product was produced in IITA-Ibadan using a laboratory-scale method previously described (Atehnkeng et al. 2008b; Senghor et al. 2020). Briefly, batches of autoclaved sorghum grain were individually inoculated with a suspension containing spores of each of the four selected atoxigenic isolates, incubated at 31 °C for 18 h, and dried in an oven (55 °C, 4 d). Equal proportions of dried grains inoculated with each isolate were mixed to constitute the product. Its quality (purity, sporulation, and composition of the active ingredient fungi) was determined as previously described (Agbetiameh et al. 2019; Senghor et al. 2020). The formulated product was placed in 2.5-kg polyethylene bags, sealed, and transported by road to Burkina Faso under export and import permits from phytosanitary authorities in Nigeria and Burkina Faso.

**Treatment of fields with the experimental biocontrol product.** The experimental product was applied in farmer fields during the 2012 and 2013 cropping seasons in Burkina Faso. Maize and groundnut fields were treated during both years in Léo, Niangoloko, and Dandé provinces. In Bogandé, only groundnut was treated during both years. The product was deployed in collaboration with members of farmers’ associations identified by INERA. All farmers voluntarily consented to conduct the trials. Farmers were advised to grow crops following their own agronomic practices without any special interventions. In general, farmers planted their preferred groundnut and maize varieties. Fields were weeded by hand or bullocks, top-dressed with urea, and earthed up (i.e., piling up soil around the base of the plants) before biocontrol application to avoid burying the product.

In both years, the product was broadcasted by hand during the second half of August (2-to-3 weeks before flowering) at the rate of 10 kg/ha. The farmers were trained by IITA and INERA on broadcasting techniques. For each treated field, a second untreated field at least 500 m away was identified. The number of biocontrol-treated and untreated fields is given in Table 4. Field sizes ranged from 0.25 ha to 5 ha. All fields were rainfall dependent. In both years, crops were harvested during the first week of November.

**Table 4:** Aflatoxin content in freshly harvested and poorly-stored maize and groundnut sampled from untreated and biocontrol-treated fields in three agroecological zones (AEZs) of Burkina Faso

Year	AEZ	Region	Crop	Treatment	N	Aflatoxin concentration (ppb)^a,b^			
						At harvest		After poor storage	
						Mean	Red. (%)^c^	Mean	Red. (%)^c^
2012	NGS	Léo	Maize	Treated	11	3	–	11	94
				Untreated	11	3		173*	
			Groundnut	Treated	15	3	96	19	87
				Untreated	15	88*		152*	
		Dandé	Maize	Treated	4	0	100	139	80
				Untreated	4	30*		691*	
			Groundnut	Treated	-	-	-	-	-
				Untreated	-	-		-	
	SGS	Niangoloko	Maize	Treated	2	0	100	5	62
				Untreated	2	2		13	
			Groundnut	Treated	16	9	74	8	90
				Untreated	16	33*		89*	
	SS	Bogandé	Groundnut	Treated	14	0	100	24	78
				Untreated	14	30*		113*	
2013	NGS	Léo	Maize	Treated	18	6	− 20	19	50
				Untreated	18	5		38	
			Groundnut	Treated	24	354	71	124	90
				Untreated	24	1,211*		1,226*	
		Dandé	Maize	Treated	17	4	20	10	57
				Untreated	17	5		23	
			Groundnut	Treated	2	0	100	0	100
				Untreated	2	23*		5	
	SGS	Niangoloko	Maize	Treated	5	3	75	8	83
				Untreated	5	12*		47*	
			Groundnut	Treated	17	14	94	44	78
				Untreated	17	246*		200*	
	SS	Bogandé	Groundnut	Treated	23	43	82	58	81
				Untreated	23	243*		299*	

NGS: Northern Guinea Savannah; SGS: Southern Guinea Savannah; SS: Sahel Savannah

^a^Mean aflatoxin values in parts per billion (ppb) are the sum of aflatoxin B1, B2, G1, and G2

^b^Means of aflatoxin values were independently compared between treated and control samples by crop in each province, per year. Values with an asterisk (*) are significantly higher from its corresponding treatment by Student’s *t*-test (α = 0.05)

^c^Percent reduction was calculated as follows: ([mean of untreated—mean of biocontrol treated]/mean of untreated) × 100

**Soil and crop sampling.** Soil samples from treated and untreated fields were collected before biocontrol application. Each soil sample (150 g) was composed of 40 to 50 sub-samples from three random locations within each field to a depth of 2 cm (Cotty 1997). For both maize and groundnut, two sets of grain samples (~ 1 kg each) were collected at harvest. One set was stored for four months in the corresponding farmer store under his/her storage conditions prior to analyses. The second set was brought directly to INERA’s Plant Pathology Laboratory and kept at 4 °C. Once all samples from both sets were gathered, these were sent to IITA-Ibadan for analyses as above.

**Experimental biocontrol product effectiveness.** The effectiveness of the experimental product was determined by using paired Student’s *t*-tests to compare densities and frequencies of *Aspergillus* section Flavi species (Table 5; Suppl. Table 2) and aflatoxin levels in crops from treated and untreated fields at harvest and after storage (Table 4). Frequencies and densities of *Aspergillus* section Flavi fungi were determined as described above. Aflatoxins were extracted and quantified as described above.

**Table 5:** Frequencies of *Aspergillus* species/morphotype distribution in soil, groundnut, and maize samples collected from biocontrol-treated and untreated fields before product application and at harvest in three agroecological zones (AEZs) of Burkina Faso in 2012 and 2013

Year	AEZ^a^	Province	Treatment	*Aspergillus* species/morphotype distribution (%)^b,c,d^															
				Maize								Groundnut							
				Soil before inoculation				Grain at harvest				Soil before inoculation				Grain at harvest			
				L	S	P	T	L	S	P	T	L	S	P	T	L	S	P	T
2012	NGS	Léo	Treated	100	0	0	0	96	4	0	0	99	1	0	0	87*	13	0	0
			Untreated	98	2	0	0	95	5	0	0	96	3	0	1	57	43*	0	0
		Dandé	Treated	100*	0	0	0	99*	1	0	0	–	–	–	–	–	–	–	–
			Untreated	93	0	3	4	78	16*	0	6	–	–	–	–	–	–	–	–
	SGS	Niangoloko	Treated	97	3	0	0	100*	0	0	0	94	5	0	1	96*	3	0	1
			Untreated	94	6	0	0	59	41*	0	0	94	5	0	1	66	33*	0	1
	SS	Bogandé	Treated	–	–	–	–	–	–	–	–	100	0	0	0	88	12	0	0
			Untreated	–	–	–	–	–	–	–	–	100	0	0	0	87	13	0	0
2013	NGS	Léo	Treated	96	3	1	0	98*	1	0	1	94	4	0	2*	99	1	0	0
			Untreated	93	1	2	4	88	12*	0	0	92	5	3	0	99	1	0	0
		Dandé	Treated	93	2	1	4	99	1	0	0	100	0	0	0	100	0	0	0
			Untreated	93	1	3	3	99	1	0	0	97	0	3	0	97	3	0	0
	SGS	Niangoloko	Treated	92	4	0	4	94	1	0	5	96	2	1	1	99*	1	0	0
			Untreated	92	5	1	2	91	9	0	0	93	1	0	6	91	9*	0	0
	SS	Bogandé	Treated	–	–	–	–	–	–	–	–	100	0	0	0	100	0	0	0
			Untreated	–	–	–	–	–	–	–	–	100	0	0	0	99	1	0	0

^a^NGS: Northern Guinea Savannah; SGS: Southern Guinea Savannah; SS: Sahel Savannah

^b^L: *Aspergillus flavus* L morphotype; S: fungi with S morphology; P: *A. parasiticus*; T: *A. tamarii*

^c^In each region, species frequencies from treated samples with an asterisk (*) significantly differed from those found in its corresponding untreated fields by Student’s *t*-test (α = 0.05)

^d^The ‘–’ character indicates that evaluations were not conducted in the corresponding year, province, crop

**Registration of the biocontrol product with regulatory authorities.** A dossier for registration of the biocontrol product with CSP/CILSS for use in maize and groundnut grown in Burkina Faso was prepared with the following information: (1) a request for registration of the formulated product; (2) a high-level summary of the information presented in the dossier; (3) a summary identifying the formulated product; (4) a section identifying the biological agents; (5) a section demonstrating the effectiveness of the product; (6) a section on the toxicology of the product; (7) a section on environmental assessment of the product; and (8) a sample of the container and the product label. A sample of the product was also submitted to CSP/CILSS. The name of the product to be registered was Aflasafe BF01.

**Selecting a distributor of the biocontrol product.** After registration of Aflasafe BF01 with CSP/CILSS, IITA and partners developed a Burkina Faso-specific commercialization strategy to determine sectors that potentially will adopt the product and the size and market demand for the different sectors. An investors’ forum was organized, and companies made expressions of interest. After an evaluation process, an investor was granted Aflasafe BF01 distribution rights through a Technology Transfer and Licensing Agreement (TTLA). This process has been described in more detail (Konlambigue et al. 2020; Bandyopadhyay et al. 2022).

**Commercial use of the biocontrol product.** For commercial usage in 2018 and 2019 in Burkina Faso, Aflasafe BF01 was produced in IITA-Ibadan and sent by road to the distributor, SAPHYTO, in Bobo Dioulasso. After 2019, the company BAMTAARE, SA produced both Aflasafe SN01 and Aflasafe BF01 in Kahone, Senegal (Ortega-Beltran et al. 2022). During the cropping seasons of 2018 and 2019, maize and groundnut farmers in Burkina Faso, under contracts with processors and agro-dealers, treated their fields with the biocontrol product. Farmers that applied the product received training and applied the product as above. Thereafter, farmers harvested, dried, and stored their crops as per their standard practices before transport to aggregation points of their farmer associations. A total of 60 maize and 12 groundnut samples (5 kg each) harvested from biocontrol-treated fields were taken from lots of 5–20 tons at the aggregation points of the different farmer associations (Table 6). For each treated sample, a 5-kg sample from lots of untreated crops was taken as an untreated sample.

**Table 6:** Aflatoxin content in biocontrol-treated and untreated commercially-produced maize and groundnut at harvest in three agroecological zones (AEZs) of Burkina Faso in 2018 and 2019

Year	Organization^a^	Crop	AEZ^b^	Treatment	n	Total aflatoxin (ppb)				Red (%)^c^
						Min	Max	Mean	Variance	
2018	Agroserv Industrie SA	Maize	SGS	Treated	6	0.6	4.2	2.2	1.6	91
				Untreated	6	2.4	90.5	23.9	1,160.9	
	UPPAL	Maize	SGS	Treated	8	0.0	2.1	1.0	0.7	95
				Untreated	8	0.5	136.0	19.2	2,230.4	
	ETW	Maize	NGS	Treated	5	1.3	4.8	3.0	0.3	54
				Untreated	5	5.0	7.9	6.4	0.3	
	USCCPA-BM	Maize	NGS	Treated	8	0.4	1.7	1.0	2.1	34
				Untreated	8	0.8	2.1	1.6	1.3	
	ETW	Groundnut	SS	Treated	6	1.5	5.3	3.5	1.0	94
				Untreated	6	2.2	194.1	60.1	3,226.6	
	AFDR	Groundnut	SS	Treated	6	0.2	2.4	1.6	2.3	94
				Untreated	6	1.8	141.8	25.9	7,097.2	
2019	FNZ	Maize	NGS	Treated	10	0.7	10.8	4.4	8.4	77
				Untreated	10	5.2	40.0	18.7	124.4	
	UPPAT	Maize	NGS	Treated	7	0.7	3.6	1.5	0.9	89
				Untreated	7	1.5	61.2	14.1	413.9	
	UPPAK	Maize	SGS	Treated	8	1.3	4.4	3.0	0.9	74
				Untreated	8	0.0	83.6	11.5	743.6	
	UPPAH	Maize	SGS	Treated	8	0.8	4.0	1.6	1.0	87
				Untreated	8	1.0	32.8	12.1	131.9	

^a^UPPAL: Union Provinciale des Professionels Agricoles de la Léraba; ETW: Ets Tangongossé Wambatié; USCCPA/BM:Union des Sociétés Coopératives pour la Commercialisation des Produits Agricoles de la Boucle du Mouhoun; AFDR: Association Formation Développement et Ruralité; FNZ: Fédération Nian Zwè; UPPAT: Union Provinciale des Professionels Agricoles du Tuy; UPPAK: Union Provinciale des Professionels Agricoles du Kénédougou; UPPAH: Union Provinciale des Professionels Agricoles du Houet

^b^SGS: Southern Guinea Savannah; NGS: Northern Guinea Savannah; SS: Sahel Savannah

^c^Percent reduction, calculated as follows: ([mean of untreated—mean of Aflasafe BF01-treated]/mean of untreated) × 100

Aflatoxins were extracted and quantified by IITA staff and partners using the USDA Grain Inspection, Packers and Stockyards Administration (GIPSA)-approved Neogen® Raptor Reader and Neogen Reveal Q + for Aflatoxin kit (Neogen Corp., Lansing, MI, USA). Briefly, each sample was blended into a powder and the blender washed with 80% ethanol between samples to prevent cross-contamination. For each sample, a 20 g sub-sample was combined with 100 ml 65% ethanol and blended for 1 min. The mixture was then filtered through Whatman No. 1 filter paper (Whatman Intl. Ltd., Maidstone, England) into a 100 ml beaker. Thereafter, 500 µl sample diluent was measured into a sample cup and 100 µl of sample filtrate was added and mixed thoroughly. Finally, 400 µl of the aliquot of the diluted sample was transferred to the cartridge of the Reveal Q + Aflatoxin kit and aflatoxin content measured with the Raptor Reader. Reveal Q + for Aflatoxin kit quantifies total aflatoxins in the range of 2 to 150 ppb. Sample filtrates with values exceeding the upper limit were diluted and reanalyzed to ensure that the quantification fell within the kit’s range.

### Rationale for using Aflasafe BF01 and Aflasafe SN01 in other countries

Mali, Niger, and Togo are countries belonging to CILSS. Stakeholders in each country have requested the use of Aflasafe products to reduce the frequent, severe contamination events in their staple crops. There is no restriction on using a product registered with CSP/CILSS in any of the 12 countries belonging to that organization. However, non-native genotypes may have reduced effectiveness in limiting aflatoxin (Bandyopadhyay et al. 2016; Mehl et al. 2012; Moral et al. 2020). Through diverse projects, hundreds of maize, groundnut, and sorghum samples were collected across Mali, Niger, and Togo to determine whether atoxigenic fungi belonging to genotypes of products registered with CSP/CILSS (i.e., Aflasafe BF01 and Aflasafe SN01) are also native there. Members belonging to the registered genotypes were found in the three countries (1–2% of the examined populations, *unpublished*) and therefore the products were tested in the three countries (see below). The natural distribution of atoxigenic *A. flavus* genotypes registered with CSP/CILSS will be reported in a separate publication.

### Biocontrol effectiveness evaluations in Mali, Niger, and Togo

**Production of Aflasafe BF01 and Aflasafe SN01 for evaluations in Mali, Niger, and Togo.** For effectiveness trials in Mali, both Aflasafe SN01 and Aflasafe BF01 were produced by BAMTAARE and sent to Institut d'Economie Rurale (IER) in Bamako, Mali in 2019, 2021, 2022, and 2023. In the case of Niger and Togo, only Aflasafe BF01 was tested. Because sending products from Senegal to either Niger or Togo was logistically complicated, the products were manufactured at IITA-Ibadan and sent to Institut National de la Recherche Agronomique du Niger (INRAN) in Niamey, Niger (in 2022), and to Institut Togolais de Recherche Agronomique (ITRA) in Lomé, Togo (in 2021 and 2022), under appropriate import and export permits. In Togo, in 2022, two formulations were tested, the regular (sterile sorghum, active ingredient spores, polymer, and dye) and an organic formulation in which the polymer (1.5 l/ton) was replaced with gum Arabic (3.5 l/ton) and the dye was omitted.

**Protocols for field effectiveness trials.** Biocontrol products were evaluated in collaboration with NGOs and their farmers operating in diverse regions together with the national agriculture research institute of each country (IER in Mali, INRAN in Niger, and ITRA in Togo). All farmers voluntarily consented orally to conduct the trials. Farmers were advised on the significance of aflatoxins and received trainings on aflatoxin management and the process of biocontrol application. General crop management protocols are mentioned above in the ‘Experimental product evaluation’ section under Burkina Faso. In all years, the product was broadcasted by hand 2-to-3 weeks before flowering at the rate of 10 kg/ha. As much as possible, for each treated field, an untreated field at least 500 m away was identified for comparison. Field sizes ranged from 0.25 ha to 5 ha. All fields were rainfall dependent.

**Fig. 4:**
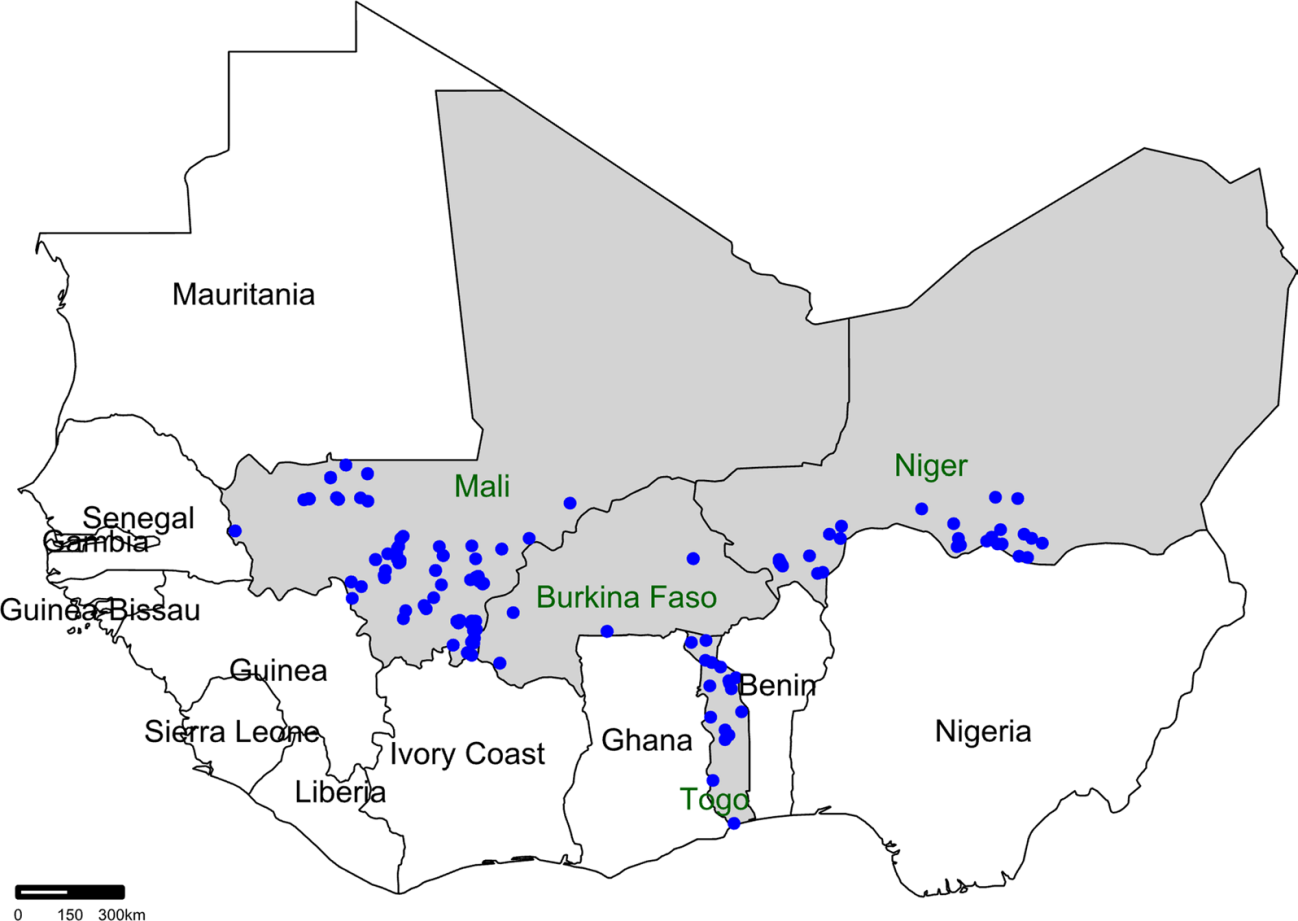
Locations in Burkina Faso, Mali, Niger, and Togo where the aflatoxin biocontrol technology was tested. Multiple locations may be represented by a single point. In Burkina Faso, there were 250 treated and 250 untreated fields. In Mali, there were 918 treated and 818 untreated fields. In Niger, there were 164 treated and 107 untreated fields. In Togo, there were 57 treated and 74 untreated fields

**Mali.** Aflasafe BF01 and Aflasafe SN01 were tested in 2019, 2021, 2022, and 2023. There were 267 treated and 239 untreated maize fields; 489 treated and 419 untreated sorghum fields; and 162 treated and 160 untreated groundnut fields (Fig. 4). In all years, crops were harvested from November to December. Aflatoxins were extracted and quantified at IER using the Neogen Raptor Reader and Neogen Reveal Q + for Aflatoxin kit, as described above.

**Niger.** Aflasafe BF01 was tested in 2022. There were 14 treated and 12 untreated maize fields; 60 treated and 36 untreated sorghum fields; and 90 treated and 59 untreated groundnut fields (Fig. 4). Crops were harvested from November to December. Aflatoxins were extracted and quantified at INRAN using the Neogen Raptor Reader and Neogen Reveal Q + for Aflatoxin kit, as described above.

**Togo.** Aflasafe BF01 and an organic formulation of Aflasafe BF01 were tested in 2021 and 2022. There were 57 treated and 74 untreated groundnut fields (Fig. 4). In both years, crops were harvested during November. Aflatoxins were extracted and quantified at ITRA using the Neogen Raptor Reader and Neogen Reveal Q + for Aflatoxin kit, as described above.

### Data analysis

Data on *Aspergillus* species/morphotype distribution and aflatoxin concentration of samples from treated and untreated fields (at harvest and after poor storage) from Burkina Faso were subjected to statistical analysis using Student’s *t*-tests (PROC TTEST; α = 0.05) with SAS software (v9.2, Cary, NC, USA). The experiments were conducted in randomized complete block designs and each farmer field was considered a replicate. The aflatoxin data for the four countries were independently analyzed using a generalized linear mixed model (McCullagh and Nelder 1989) under the assumption of a binomial distribution (Whitaker et al. 1996) for each crop by country combination. We tested whether there was a significant treatment × year interaction effect and when that was the case, we fitted a model for each year separately, where treatment was considered as the fixed effect and village as the random effect. Predicted means and the associated standard errors derived from the generalized linear mixed model were computed. We compared and ranked predicted means using Tukey’s Honest Significant Difference Test. All analyses were carried out in R (R Core Team, 2024) using lme4 (Bates et al. 2015) for fitting the linear mixed models.

## Results

### Biocontrol product development and effectiveness in Burkina Faso

**Fungal densities.** Fungi belonging to *Aspergillus* section Flavi were detected in all examined crop and soil samples collected throughout Burkina Faso. Mean CFU/g of sample values varied among and within AEZs. Densities in maize and groundnut soils ranged from 31 CFU/g to 60,000 CFU/g and from 31 CFU/g to 4,200 CFU/g, respectively (Suppl. Table 1). Densities in grains ranged from 3 CFU/g to 600,000 CFU/g for maize and from 3 CFU/g to 400,000 CFU/g in groundnut (Suppl. Table 1). No trend was detected where one AEZ consistently harbored more *Aspergillus* spp. than any other.

***Aspergillus* section Flavi incidence.** The *A. flavus* L morphotype dominated all substrates (Table 1). In 47% of the soils and 75% of the grains, no other *Aspergillus* fungus was detected. For maize grain, in any province, the maximum percentage of *Aspergillus* section Flavi other than the L morphotype was 5%. Similar proportions were detected in maize soils of Sahel Savannah (SS) provinces. However, higher proportions of fungi with S morphology (up to 40%) and *A. tamarii* (up to 27%) were detected in maize soils of certain provinces of Northern Guinea Savannah (NGS) and Southern Guinea Savannah (SGS) (Table 1). *A. parasiticus* was not detected in any maize grain or soil sample. For groundnut soil, the L morphotype dominated in SS (98 to 100%), while variable community structures were detected in the two other AEZs (up to 47% of fungi with S morphology, Table 1). *A. parasiticus* was not detected in groundnut soils. Regarding groundnut grain, communities were completely dominated by the L morphotype (100%) across provinces except in a province of NGS where *A. parasiticus* composed 20% of the fungi and in a province of SS (fungi with S morphology composed 2%). *A. tamarii* was not detected in groundnut grain samples.

**Aflatoxin-producing abilities of *A. flavus* L morphotype isolates.** There were 2826 *A. flavus* L morphotype isolates recovered, and all were evaluated for aflatoxin-producing ability (Table 2). In each province, the percentage of atoxigenic isolates in maize ranged from 7 to 40% in soil and from 0 to 47% in grain. In groundnut, per province, atoxigenic isolates ranged from 0 to 25% in soil and from 0 to 23% in grain. Aflatoxin-producing potentials of toxigenic fungi were variable among provinces, AEZs, substrates, and crops (Table 2). Nearly 340 atoxigenic fungal germplasms native to Burkina Faso were detected with 239 isolates recovered from maize substrates and 98 from groundnut substrates.

**Molecular studies of atoxigenic fungi.** SSR analysis of the 146 atoxigenic *A. flavus* isolates revealed 134 haplotypes with variable CAPs results and mating-type idiomorphs (Fig. 2A, B). Some isolates amplified each of the 32 markers while others amplified from 2 to 31 markers. The four atoxigenic isolates selected to constitute the product Aflasafe BF01 (Table 3; Fig. 2) did not amplify several of the 32 CAPs markers (Fig. 3). Isolate M110-7 amplified only one marker of the sugar cluster. Isolate G018-2 amplified few markers, including some in the aflatoxin cluster but none of the CPA markers. Both M011-8 and M109-2 amplified all aflatoxin markers, a few in the sub-telomere region, and missed a CPA marker. Aflatoxin gene cluster deletions in M011-8 and M109-2 correspond to pattern A, G018-2 has pattern D deletions, and M110-7 has pattern F deletions (Fig. 2B) as per the descriptions of Adhikari et al. (2016). B, C, and E deletion patterns were not found among the examined set of atoxigenic isolates from Burkina Faso.

Isolates M011-8, M109-2, and G018-2 possess the *MAT1-2* idiomorph while M110-7 possess the *MAT1-1* idiomorph. Deletion pattern was not associated with a particular idiomorph. Isolates possessing either *MAT1-1* or *MAT1-2* were found within each deletion pattern outlined above (Fig. 2B).

**VCA.** Complementary tester pairs were developed for each of the four atoxigenic isolates selected to compose Aflasafe BF01. VCG grouping concurred with that revealed by SSRs, and none of the atoxigenic isolates had vegetative compatibility with the isolates with toxigenic capability (*data not shown*). The VCGs to which the atoxigenic isolates composing Aflasafe BF01 belong are named AAV-BF011 (AAV: African *Aspergillus* VCG), AAV-BF018, AAV-BF109, and AAV-BF110 (Table 3).

**Quality control of Aflasafe BF01.** All examined Aflasafe BF01 batches yielded 100% of carrier grains colonized by *A. flavus*. The recovered *A. flavus* fungi were solely composed of the Aflasafe BF01 genotypes. There were no other microorganisms recovered in any of the grains. Each Aflasafe BF01 genotype was found on 25 ± 5% carrier grains of the examined batches. Spore yield was, on average, 3500 ± 300 CFU/g of product.

**Fungal communities before and after application of Aflasafe BF01.** In general, fungal densities in soils before biocontrol application were similar in both maize and groundnut fields (Suppl. Table 2). There were only two cases, both in Niangoloko in 2013, in which fungal densities differed (*P* < 0.05), with higher densities in maize soils to be untreated and groundnut soils to be treated. Regarding fungal densities in grains at harvest, higher (*P* < 0.05) densities were detected in treated grains of four of the six maize province-year evaluations, and in treated kernels of three of the seven groundnut province-year evaluations (Suppl. Table 2). There was one case in which higher densities (*P* < 0.05) were detected in untreated groundnut kernels at harvest.

**Fig. 5:**
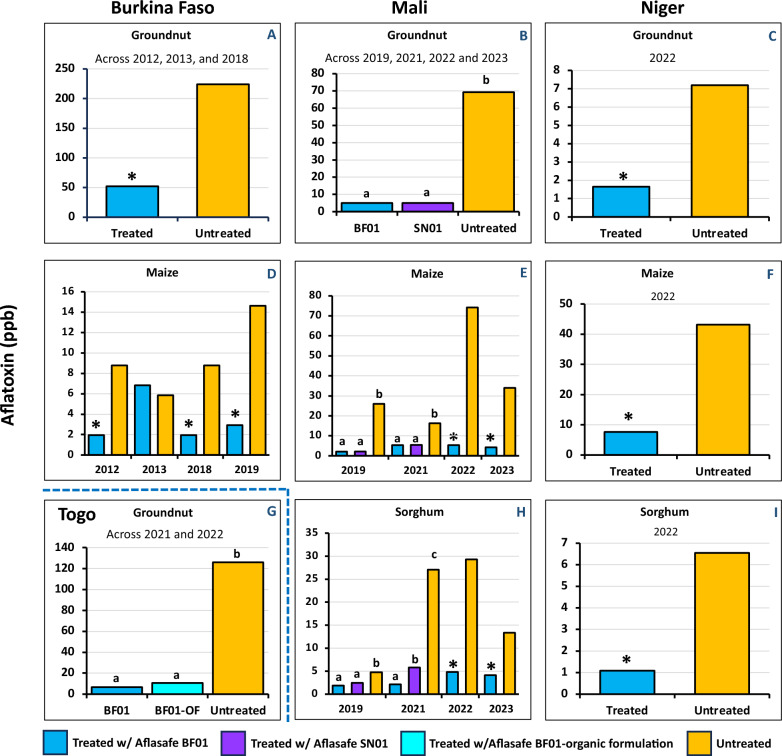
Total aflatoxin content in maize, groundnut, and sorghum from treated and untreated fields in Burkina Faso, Mali, Niger, and Togo, during multiple years. In Burkina Faso and Niger, only Aflasafe BF01 was tested. In Mali, both Aflasafe BF01 and Aflasafe SN01 were tested. In Togo, Aflasafe BF01 and an organic formulation of Aflasafe BF01 were tested. Bars of treated samples with an asterisk (*) or with different smallcase letters (when two formulations were tested) significantly differed from those in their corresponding untreated crops (Tukey HSD; α = 0.05). In Burkina Faso, sorghum was not treated. In Togo, only groundnut was treated

**Aflatoxin concentrations in treated and untreated crops.** Overall, significantly lower aflatoxin content was detected both at harvest and after poor storage in grains from fields treated with Aflasafe BF01 (Table 4). At harvest, both maize and groundnut from treated fields contained up to 100% less aflatoxins compared to grains from untreated fields. Only in one province during 2013, Leo, maize at harvest from treated fields had higher (6 ppb) aflatoxin content than maize from untreated fields (5 ppb), but there were no significant differences between treatments. Total aflatoxin content in treated grains ranged from 0 to 6 ppb in maize, and from 0 to 354 ppb in groundnut. In contrast, aflatoxin content ranged from 2 to 30 ppb in untreated maize grains and from 23 ppb to 1211 ppb in untreated groundnut grains (Table 4). In a separate analysis combining results of each year, significantly less aflatoxin was found in the treated grains in all crop-years combinations (Fig. 5A, D), except for maize in 2013 where treated and untreated grains had similar, low aflatoxin levels.

Under poor storage conditions, grains from treated fields contained 50 to 100% less aflatoxins than grains from untreated fields (Table 4). The total aflatoxin content ranged from 5 to 139 ppb in treated maize, and from 0 to 124 ppb in treated groundnut. In untreated maize, aflatoxins ranged from 13 to 691 ppb, while in untreated groundnut aflatoxins ranged from 5 to 1226 ppb.

***Aspergillus* fungi before and after application of Aflasafe BF01.** The *A. flavus* L morphotype largely dominated (range = 92 to 100%) all examined soils regardless of crop, treatment, province, and year (Table 5). Frequencies of the highly toxigenic fungi with S morphology and *A. parasiticus* were never greater than 6% and 3%, respectively. *A. tamarii* was rarely found in soils during 2012 while in 2013 its frequencies ranged from 0 to 6% (Table 5). At harvest, the *A. flavus* L morphotype continued to dominate both treated and untreated grains of both maize and groundnut but frequencies of fungi with S morphology increased considerably, particularly in untreated fields (Table 5). In many cases, there were higher (*P* < 0.05) frequencies of fungi with S morphology in untreated grains of both crops. *A. parasiticus* was never found in maize or groundnut grains at harvest, regardless of treatment, while *A. tamarii* was detected only in a few provinces during each year, sometimes in treated and others in untreated grains.

**Aflatoxin concentrations in treated and untreated commercial crops at harvest.** Commercially grown, treated maize and groundnut always had lower aflatoxin content than untreated grains (Table 6). Aflatoxin in treated maize ranged from 0 to 10.8 ppb while in groundnut aflatoxin ranged from 0.2 to 5.3 ppb. On the other hand, untreated maize had an aflatoxin range of 0.5 to 136 ppb, while untreated groundnut had an aflatoxin range of 1.8 to 194 ppb (Table 6). Maximum variance of aflatoxin in treated grains was 2.3 while in untreated grains was 7097 (Table 6).

### Biocontrol product effectiveness in Mali, Niger, and Togo

**Mali.** A significant treatment × year interaction effect was found in the maize and sorghum trials, but not in the groundnut ones (Table 7). The analysis combining the groundnut values across years revealed significantly less (*P* < 0.05) aflatoxin in treated than in untreated grains and no differences in reduction between the two tested products (Fig. 5B). In maize trials, in all cases, treated crops had lower (*P* < 0.05) aflatoxin content than untreated crops, and in the two years when the two biocontrol products were tested (2019 and 2021), there were no differences (*P* > 0.05) between them (Fig. 5E). Similarly, in the sorghum trials, there was less (*P* < 0.05) aflatoxin in treated crops, but in one of the two years when the two biocontrol products were tested, the crops treated with Aflasafe BF01 had less (*P* < 0.05) aflatoxin than Aflasafe SN01-treated crops (Fig. 5H). In treated crops, regardless of the product used, the average aflatoxin content ranged from 1.8 to 5.8 ppb (Fig. 5). On an average, the lowest aflatoxin content in untreated crops in Mali occurred in sorghum, ranging from 6 to 43 ppb. The highest aflatoxin content in untreated maize and untreated groundnut was 74 ppb (in 2022) and 69 ppb (avg. of four years), respectively.

**Table 7:** Analysis of variance for mixed models of data on total aflatoxin values from biocontrol treated and untreated crops in four different countries during multiple years

Crop	Country	Effect	Df	*P*-value^a^
Groundnut	Burkina Faso	Aflasafe	1	< 0.0001
		Year	2	< 0.0001
		Aflasafe × Year	2	0.3270
	Mali	Aflasafe	2	0.0000
		Year	1	0.1354
		Aflasafe × Year	2	0.1859
	Niger	Aflasafe	1	< 0.0001
	Togo	Aflasafe	2	0.0000
		Year	1	0.0225
		Aflasafe × Year	1	0.9801
Maize	Burkina Faso	Aflasafe	1	< 0.0001
		Year	3	0.3849
		Aflasafe × Year	3	< 0.0001
	Mali	Aflasafe	2	< 0.0001
		Year	3	0.0020
		Aflasafe × Year	4	< 0.0001
	Niger	Aflasafe	1	< 0.0001
Sorghum	Mali	Aflasafe	2	< 0.0001
		Year	3	< 0.0001
		Aflasafe × Year	4	< 0.0001
	Niger	Aflasafe	1	< 0.0001

^a^Data were analyzed under the assumption of a binomial distribution for each crop by country combination. When there was significant treatment × year interaction effect, a model for each year was fitted separately, where treatment was considered as fixed effect and village as random effect. Predicted means were compared and ranked using Tukey’s Honest Significant Difference Test

**Niger.** Significantly less aflatoxin (*P* < 0.05) accumulated in all treated crops (range = 1.1 to 7.6 ppb) compared to untreated crops (range = 6.5 to 43.1 ppb) (Fig. 5C, F, I). Treated groundnut and treated sorghum had, on an average, less than 2 ppb total aflatoxin.

**Togo.** Groundnut was the only crop treated in Togo (Fig. 5G) for two years (2021 and 2022). There was no significant treatment × year interaction found (Table 7), therefore results of both years were combined. Two Aflasafe BF01 formulations were tested, the original and an organic one. Treated crops had lower (*P* < 0.05) aflatoxin content than untreated crops, and there were no differences (*P* > 0.05) between the two formulations (Fig. 5G). Aflatoxin in untreated groundnut was, on average, 126 ppb, while crops treated with the original formulation had 6.6 ppb and those treated with the organic formulation had 10.7 ppb total aflatoxin.

## Discussion

The current study reports (i) the development, testing, registration, and scaling of a biocontrol product for aflatoxin mitigation in maize and groundnut in Burkina Faso; and (ii) efforts to have the product tested at scale in maize, groundnut, and sorghum grown in Mali, Niger, and Togo. In addition, an aflatoxin biocontrol product developed for use in Senegal was tested in Mali, and an organic aflatoxin biocontrol formulation was tested in Togo. This is the first report of the effectiveness of atoxigenic-based aflatoxin biocontrol products in four countries. Previously, the effectiveness of an aflatoxin biocontrol product in two countries was reported (Senghor et al. 2021; Ortega-Beltran et al. 2022). In the current study, a country-specific biocontrol product initially developed for Burkina Faso was also evaluated in three other countries (Mali, Niger, and Togo). Also, the aflatoxin biocontrol effectiveness is reported for the first time in sorghum, a crop that was considered relatively safe from aflatoxin but recently has been affected by contamination events, requiring aflatoxin management strategies. Aflatoxin was analyzed in grains obtained from a large number of fields (1389 treated and 1249 untreated) managed by smallholder farmers. The use of aflatoxin biocontrol products in all four countries resulted in substantial aflatoxin reductions (up to 100% less) in treated crops compared to untreated crops (Tables 4, 6, 7; Fig. 5). When two formulations were tested, similar low aflatoxin content was detected in the treated crops, except in one case (sorghum in Mali in 2021), although use of either formulation resulted in less aflatoxin than the corresponding untreated crops. The current study first describes research efforts targeting aflatoxin biocontrol for use in one country and then how those efforts were capitalized for rapid use of the technology in other countries. Starting individual programs in countries from the ground up, when the technology is readily available in neighboring countries, would have resulted in lost opportunities and farmers having to wait several years, if not decades, for products to be developed, validated, and scaled for them to use to protect their crops.

Countries in SSA are repeatedly affected by aflatoxin contamination events. Farmers growing aflatoxin-prone crops in the sub-region need aflatoxin management tools. In 2010, the Austrian Development Agency provided funds for IITA, USDA-ARS, and INERA to develop the aflatoxin biocontrol technology for use in Burkina Faso. The sample collection (Fig. 1) and fungal characterization (Tables 1, 2; Figs. 2, 3) allowed detecting several atoxigenic isolates of *A. flavus* for constituting a product (Table 3) for large-scale field testing, as required by the regulator CSP/CILSS. Application in farmers’ fields across Burkina Faso occurred in 2012 and 2013, and tests for aflatoxin revealed that the use of the experimental product resulted in significantly less aflatoxin in treated crops compared to untreated crops (Table 4; Fig. 5). The results of the effectiveness trials, along with other information, were used to prepare a dossier for registration of Aflasafe BF01 with CSP/CILSS. CSP/CILSS is the regulatory agency for pesticides and bioprotectants in 13 countries of the Sahel region, including Burkina Faso. After evaluation of the dossier, CSP/CILSS registered Aflasafe BF01 in May 2017, for aflatoxin control in maize and groundnut. The availability of Aflasafe BF01 for use at scale can allow farmers and consumers in Burkina Faso to produce, and have access to, aflatoxin-compliant crops, respectively. Registration also allows the product to be used in any of the 12 other CILSS countries.

The examination of fungal communities revealed that the *A. flavus* L morphotype dominated both soils and crops (Table 1). Fungi with S morphology were rarely found in maize and groundnut grains even though this group thrives in dry, hot environments (Agbetiameh et al. 2018; Atehnkeng et al. 2008a; Diedhiou et al. 2011; Donner et al. 2009). In addition, *A. parasiticus* was not detected in any groundnut or maize soil and was found only in groundnut kernels from one province. *A. parasiticus* is common in groundnut-producing regions across the globe (Klich 2007) but results from the current study provide additional evidence that this species is rarely associated with groundnut and other crops in West Africa (Agbetiameh et al. 2018; Diedhiou et al. 2011; Donner et al. 2009; Ezekiel et al. 2019; Senghor et al. 2020).

Aflatoxin-producing isolates of *A. flavus* (88%) had variable toxigenic potentials with communities from certain provinces producing on an average up to 220,000 ppb in maize fermentations (Table 2). The remaining *A. flavus* fungi were atoxigenic and found in all provinces, although in some soils or grains of certain provinces were not detected (Table 2).

Several aflatoxin biocontrol products under the trade name Aflasafe have been developed by IITA and USDA-ARS, in collaboration with local and international organizations (Bandyopadhyay et al. 2022). The active ingredients of Aflasafe products are atoxigenic *A. flavus* genotypes native to the target country. Atoxigenic *A. flavus* genotypes are relatively common in all areas where those have been sought (Alaniz Zanon et al. 2016; Cotty 1997; Dorner 2004; Mauro et al. 2015; Probst et al. 2014; Savi et al. 2020; Wei et al. 2014), as in the current study (Table 2). There were 136 atoxigenic genotypes among the 148 atoxigenic isolates, revealed by the 13 SSR markers (Fig. 2A, B). In addition, the nature of atoxigenicity of each genotype was determined by looking for indels in genes necessary for aflatoxin production (Callicott and Cotty 2015). Of the eight patterns of defects in genes responsible for aflatoxin and CPA formation reported by Adhikari et al. (2016), three (A, D, and F) were found in the current study (Fig. 2B).

The reasons for atoxigenicity for those that amplified all aflatoxin markers (pattern A) but did not produce aflatoxins may be due to the presence of SNPs conferring atoxigenicity and/or deletions in areas not covered by CAPs markers. From this pool of atoxigenic genotypes, four isolates were selected to constitute the experimental biocontrol product. Two isolates, M011-8 and M109-2, have pattern A, while G018-2 and M110-7 have patterns D and F, respectively (Fig. 2B). Three of the isolates (M011-8, M109-2, and M110-7) belong to atoxigenic VCGs/SSR groups which are relatively common across the sampled regions (Table 3; Fig. 2A). The fourth isolate, G018-2, belongs to an atoxigenic VCG that was less common but possesses complete deletions in aflatoxin and CPA gene clusters (Table 3). Also, G018-2 was initially isolated from groundnut while the other three active ingredients were isolated from maize. A comparative analyses of aflatoxin gene clusters of these four isolates has been published (Adhikari et al. 2016). It should be investigated if contemporary atoxigenic fungal communities across Burkina Faso possess other deletion patterns reported before, or yet to be described. Three of the isolates, M011-8, M109-2, and G018-2 possess the *MAT1-2* idiomorph while M110-7 possess the *MAT1-1* idiomorph. While use of mating-type information has been recommended for selecting biocontrol strains (Molo et al. 2019), both biocontrol products used in the current study were highly effective despite not knowing mating-type information when active ingredients were selected for the products. A few recent studies have suggested assessing the mating-type profiles of aflatoxin-producers in fields before deploying biocontrol products (Chang 2022; Moore 2022; Moore et al. 2017). Nevertheless, effective aflatoxin control (Fig. 5; Table 7) was achieved at scale, in the four countries, regardless of prior knowledge of mating-type profiles in the fields that were treated.

Because of variability in aflatoxin contamination in untreated crops, testing of atoxigenic-based aflatoxin biocontrol products must be conducted in multiple fields, during multiple years, and preferably under the conditions that the farmers face (Agbetiameh et al. 2019; Ezekiel et al. 2019; Senghor et al. 2020). Testing a technology in that manner allows determining if it is of practical use. The trials conducted in Burkina Faso in 222 groundnut and 114 maize fields for two years revealed that the product significantly limited aflatoxin across the tested AEZs (Table 4). There was only one instance—groundnut in Burkina Faso—where the average aflatoxin content in treated crops was considered high for human consumption (avg. across years = 52 ppb). Several factors could have influenced biocontrol being unable to reduce aflatoxin levels to safer limits in some treated fields during the initial testing years (2012 and 2013), including improper timing or dosage of application, environmental conditions not supporting the required sporulation of the biocontrol fungi, and the presence of highly toxigenic fungi in those treated fields. However, the reduction compared to untreated crops (avg. across years = 224 ppb) was 76%, a statistically significant (*P* < 0.05) decrease (Fig. 5A).

In addition to the field effectiveness, the product was found to confer protection even if the crops were stored under sub-optimal conditions. Aflatoxin reductions ranged from 57 to 100% in the treated crops compared to the untreated crops after 4-months of storage (Table 4). This contrasts the conclusions of Gressel and Polturak (2018) and Kinyungu et al. (2019) that pre-harvest application of biocontrol does not offer post-harvest benefits. Kinyungu et al. based their conclusions on experiments in which treated and untreated crops were incubated in laboratory conditions. However, sub-optimal crop storage involves a different environmental context, characterized by less conducive conditions to continuous aflatoxin production. While laboratory studies may raise theoretical concerns regarding the potential absence of post-harvest benefits, the practical implementation of aflatoxin biocontrol has proven effective in mitigating aflatoxin contamination in real-world agricultural contexts, both at the pre- and post-harvest stage, as in the current and other studies (Atehnkeng et al. 2022; Bandyopadhyay et al. 2019; Senghor et al. 2021).

After a competitive process, the company SAPHYTO was selected as the distributor of Aflasafe BF01 (Bandyopadhyay et al. 2022; Konlambigue et al. 2020). SAPHYTO (i) markets the product to several farmers’ organizations and extension service providers, (ii) provides training to farmers for correctly using the technology, and (iii) demonstrates biocontrol effectiveness by quantifying aflatoxin at harvest using *in-situ* testing systems as described by Ortega-Beltran et al. (2022). At the aggregation points, the aflatoxin quantification in samples collected from lots made of treated and untreated crops revealed lower aflatoxin in treated lots (Table 6). All treated crop-year-AEZ combinations had low average aflatoxin content, whereas only two of the nine organizations produced crops with low average aflatoxin in the absence of treatment. Either fungal communities in fields where those crops were grown had low aflatoxin-producing potentials or the environmental conditions did not favor aflatoxin formation. However, the average aflatoxin range in the rest of the untreated crop-year-AEZ combinations was substantially higher and extremely variable (Table 6).

The low aflatoxin concentrations found in the samples from treated, commercially grown crops in Burkina Faso (Fig. 5D) shows that private sector-led distribution efforts and training of farmers on biocontrol product use results in crops with low aflatoxin. Appropriate awareness raising and training on the use of aflatoxin biocontrol products are a must for effective aflatoxin protection (Hoffmann et al. 2018, 2022). The key role of the private sector in reducing aflatoxin contamination through biocontrol has also been reported in Nigeria, Senegal, and The Gambia (Ola et al. 2022; Ortega-Beltran et al. 2022; Senghor et al. 2021).

Published reports of the effectiveness of aflatoxin biocontrol in several African countries, in addition to white papers and presentations in meetings coordinated by various organizations (e.g., Partnership for Aflatoxin Control in Africa) where benefits, challenges, and opportunities of the technology have been discussed has resulted in diverse stakeholders demanding the introduction of the biocontrol technology in their countries. However, funding acquisition is required for development of biocontrol products. When this occurred for projects in Mali, Niger, and Togo, it was possible to test the aflatoxin biocontrol technology in each of those three countries. The respective national research organizations IER, INRAN, and ITRA, in collaboration with IITA, identified farmer organizations and Non-Governmental Organizations (NGOs) affected by aflatoxin contamination and interested in testing the technology. Several of the training activities, both online and in person, took place during the COVID-19 lockdowns. In turn, the NGOs in each country oversaw the training of their farmers in the correct usage of the products and the collection of the samples for the analyses. In some cases, it was not possible to obtain equal numbers of treated and untreated samples (Fig. 4). The results, however, clearly indicate that the aflatoxin biocontrol technology allowed production of safe crops in Mali, Niger, and Togo, in addition to Burkina Faso (Fig. 5, Table 7). Untreated groundnut and sorghum in Niger, and untreated sorghum in Mali in 2019 were the only untreated crop-country-year combinations that had low aflatoxin levels (Fig. 5C, F, H). The rest of the comparisons yielded unsafe aflatoxin content in the untreated crops demonstrating the widespread occurrence of aflatoxins in these countries’ food systems.

Several studies have reported the testing of aflatoxin biocontrol products in controlled environments and a limited number of fields (Alaniz Zanon et al. 2016; Molo et al. 2019; Weaver and Abbas 2019; Weaver et al. 2015). A relatively recent study conducted comparison across four US states involving the two US Environmental Protection Agency (US-EPA) approved biocontrol products in microplot trials and evaluated the influences of biocontrol application on native *Aspergillus* community structure (Molo et al. 2022). However, it is important to note that testing the effectiveness of atoxigenic biocontrol products requires assessments across multiple fields over several years. This is because a large portion of the untreated crops will not be naturally contaminated, as seen in the current study (Tables 4, 6; Fig. 5) or there could be potential biocontrol interference because of the proximity of treated and untreated fields.

Several mechanisms have been described through which atoxigenic isolates reduce the quantities of aflatoxins in crops. These include direct influences on regulation of aflatoxin biosynthesis (Cotty and Bayman 1993; Hua et al. 2019; Huang et al. 2011), direct competition during invasion of crop tissues (Mehl and Cotty 2010), direct degradation of aflatoxins (Maxwell et al. 2021), and modification of fungal populations throughout the environment where crops are produced (Cotty et al. 2007). Most described mechanisms require that the atoxigenic active ingredient isolates interact with either aflatoxin-producers or with aflatoxins in crop tissues. The only mechanism practically demonstrated repeatedly to function under crop production is modification of fungal population structure (Atehnkeng et al. 2022; Cotty 2007). Aflatoxin concentrations decrease as the incidence of atoxigenic isolates increase (Cotty 2007). This mechanism is also responsible for long-term and area-wide benefits of atoxigenic strain use (Cotty 2006). The current study detected beneficial influences by Aflasafe BF01 on fungal population structure as increases of L morphotype incidence and decreased incidence of high aflatoxin-producing fungi with S morphology (Table 5). Follow-up studies to quantify multi-season persistence and long-term dispersal of Aflasafe BF01 active ingredients are needed to determine the most cost-effective frequencies of applications by smallholder farmers in West Africa.

In conclusion, the successful multi-country efforts reported in the current study underscore the significance of project funding, collaborative research, and technology transfer in addressing aflatoxin contamination, a persistent, serious challenge across SSA. By leveraging existing knowledge, partners’ ecosystem and infrastructure, the development and deployment of aflatoxin biocontrol technology were expedited, offering a timely solution to farmers across multiple countries. Moreover, the involvement of private sector entities in product distribution and farmer training highlights the importance of stakeholder engagement and capacity building in scaling up aflatoxin management strategies.

Moving forward, sustaining these efforts will require continued collaboration among researchers, policymakers, the donor community, and industry stakeholders to ensure widespread adoption and impact, along with appropriate incentive mechanisms for farmers producing aflatoxin-safe crops and industries committed to provide management tools to farmers (Narayan and Geyer 2022). Additional research may focus on refining biocontrol formulations, optimizing application methods, and expanding the range of crops and regions covered. Further, efforts to secure funding and support for aflatoxin management initiatives remain critical to addressing this complex agricultural challenge comprehensively. Overall, the findings presented here offer a promising pathway toward enhancing food safety and security in Burkina Faso, Mali, Niger, Togo, and beyond. We call upon governments, donors, private sectors, development organizations, and other stakeholders to act and promote emphasizing the potential of biocontrol technologies as sustainable solutions to aflatoxin contamination.

## Supplementary Information

Additional file 1.

## Data Availability

The datasets used and/or analysed during the current study are available from the corresponding authors on reasonable request.

## Abbreviations

AAV: African *Aspergillus* VCG
AEZ: Agroecological zones
APHIS: USDA’s Animal and Plant Health Inspection Service
CAPs: Cluster amplification patterns
CFU: Colony-forming units
CPA: Cyclopiazonic acid
CSP: Comité Sahélien des Pesticides
CILSS: Comité permanent Inter-Etats de Lutte contre la Sécheresse dans le Sahel
GIPSA: USDA Grain Inspection, Packers and Stockyards Administration
IER: Institut d’Economie Rurale
IITA: International Institute of Tropical Agriculture
INERA: Institut de l'Environnement et de Recherches Agricoles
INRAN: Institut National de la Recherche Agronomique du Niger
ITRA: Institut Togolais de Recherche Agronomique
NGO: Non-Governmental Organization
NGS: Northern Guinea Savannah
ppb: Parts per billion
SGS: Southern Guinea Savannah
SS: Sahel Savannah
SSA: sub-Saharan Africa
SSR: Simple sequence repeat
TTLA: Technology Transfer and Licensing Agreement
USDA-ARS: United States Department of Agriculture—Agricultural Research Service
US-EPA: United States Environmental Protection Agency
VCA: Vegetative compatibility analyses
VCG: Vegetative compatibility group
