# Supplementary material for: Mitigation of aflatoxin contamination of maize, groundnut, and sorghum by commercial biocontrol products in farmers’ fields across Burkina Faso, Mali, Niger, and Togo
Source: CABI Agric Biosci. 2024 Nov 11;5(1):106. doi: 10.1186/s43170-024-00313-3 (PMC11554699; doi:10.1186/s43170-024-00313-3)
Supplement: Supplementary file 1 — Additional file 1. [file 43170_2024_313_MOESM1_ESM.docx]

**Supplementary Table 1**. Densities of *Aspergillus* section *Flavi* fungi in maize and groundnut soil and grain samples collected in 2010 across 16 provinces in three agroecological zones (AEZ) of Burkina Faso

| AEZ^a^ | Province | CFU/g^b^ | | | | | | | | | | | | |
| --- | --- | --- | --- | --- | --- | --- | --- | --- | --- | --- | --- | --- | --- | --- |
|  |  | Maize | | | | | |  | Groundnut | | | | | |
|  |  |  | Soil | |  | Grain | |  |  | Soil | |  | Grain | |
|  |  | n^c^ | Mean | Range |  | Mean | Range |  | n^c^ | Mean | Range |  | Mean | Range |
| NGS | Balé | 4 | 263 | 67 - 350 |  | 94 | 17 - 225 |  | 2 | 175 | 100 - 250 |  | 202 | 3 - 4,00 |
|  | Boulgou | 5 | 1,155 | 125 - 4,500 |  | 1,261 | 35 - 60,00 |  | 3 | 407 | 38 - 900 |  | 393 | 56 - 1,000 |
|  | Houet | 6 | 1,090 | 200 - 3,333 |  | 90,827 | 4 - 600,000 |  | 4 | 1,300 | 900 - 2,000 |  | 37 | 17 - 56 |
|  | Kénédougou | 1 | 850 | 700 - 1,000 |  | 33 | 6 - 63 |  | 3 | 82 | 50 - 113 |  | 702 | 3 - 1,400 |
|  | Kouritenga | 5 | 2,733 | 400 - 6,000 |  | 243 | 4 - 667 |  | 3 | 421 | 63 - 500 |  | 239 | 167 - 300 |
|  |  |  |  |  |  |  |  |  |  |  |  |  |  |  |
| SGS | Cascades | 1 | 500 | - |  | 22 | - |  | 1 | 800 | - |  | 1,429 | - |
|  | Comoé | 5 | 713 | 31 - 1,286 |  | 44 | 3 - 88 |  | 4 | 661 | 31 - 2,000 |  | 5,149 | 47 - 20,000 |
|  |  |  |  |  |  |  |  |  |  |  |  |  |  |  |
| SS | Bazéga | 2 | 1,200 | 1,200 - 1,800 |  | 43 | 10 - 63 |  | 2 | 363 | 125 - 600 |  | 1,400 | 800 - 2,000 |
|  | Boulkiemdé | 4 | 18,592 | 500 - 60,000 |  | 29 | 4 - 44 |  | 4 | 883 | 50 - 2,000 |  | 99 | 10 - 250 |
|  | Gnagna | 5 | 3,550 | 1,000 - 8,000 |  | 59,383 | 83 - 210,000 |  | 5 | 1,610 | 350 - 3,600 |  | 161,167 | 3 - 400,000 |
|  | Gourma | 7 | 1,492 | 125 - 4,500 |  | 22 | 5 - 50 |  | 7 | 492 | 67 - 1,200 |  | 66,824 | 3 - 400,000 |
|  | Kadiogo | 4 | 4,100 | 800 - 9,000 |  | 36 | 4 - 56 |  | - | - | - |  | - | - |
|  | Komandjari | 4 | 2,404 | 450 - 4,500 |  | 30,276 | 5 - 120,000 |  | 5 | 831 | 83 - 2,000 |  | 821 | 12 - 1,800 |
|  | Kourwéogo | 1 | 1,200 |  |  | 44 | - |  | - | - | - |  | - | - |
|  | Oubritenga | 5 | 1,779 | 64 - 3,600 |  | 54,192 | 3 - 270,000 |  | 5 | 1,527 | 83 - 4,200 |  | 344 | 19 - 800 |
|  | Sanmatenga | 2 | 3,200 | 1,600 - 4,800 |  | 30 | 29 - 31 |  | 3 | 150 | - |  | 100 | - |

^a^ NGS: Northern Guinea Savannah; SGS: Southern Guinea Savannah; SS: Sahel Savannah

^b^ Colony-forming units per g of sample. Values were obtained from soil and crops samples by dilution plating technique

^c^ For each crop, grain and soil samples were obtained from the same field

**Supplementary Table 2** Densities of *Aspergillus* section Flavi in soil, groundnut, and maize collected from untreated and biocontrol treated before biocontrol application and at harvest in three agroecological zones of Burkina Faso in 2012 and 2013

| **Region^a^** | **Province** | **Treatment** | **CFU/g^b,c^** | | | | | | | | | | | | | | |
| --- | --- | --- | --- | --- | --- | --- | --- | --- | --- | --- | --- | --- | --- | --- | --- | --- | --- |
|  |  |  | **Maize** | | | | | | |  | **Groundnut** | | | | | | |
|  |  |  | **2012** | | |  | **2013** | | |  | **2012** | | |  | **2013** | | |
|  |  |  | **n** | **Soil before inoculation** | **Grain at harvest** |  | **n** | **Soil before inoculation** | **Grain at harvest** |  | **n** | **Soil before inoculation** | **Grain at harvest** |  | **n** | **Soil before inoculation** | **Grain at harvest** |
| NGS | Léo | Treated | 11 | 1,163 | 11,293* |  | 20 | 756 | 1,869* |  | 15 | 822 | 2,572 |  | 20 | 357 | 33,238 |
|  |  | Untreated | 11 | 863 | 5,534 |  | 20 | 485 | 177 |  | 15 | 220 | 6,845* |  | 20 | 330 | 12,075 |
|  |  |  |  |  |  |  |  |  |  |  |  |  |  |  |  |  |  |
|  | Dandé^d^ | Treated | 4 | 347 | 3,925 |  | 18 | 450 | 144 * |  | - | - | - |  | 2 | 325 | 900 |
|  |  | Untreated | 4 | 288 | 2,925 |  | 18 | 827 | 17 |  | - | - | - |  | 2 | 250 | 925 |
|  |  |  |  |  |  |  |  |  |  |  |  |  |  |  |  |  |  |
| SGS | Niangoloko | Treated | 2 | 525 | 4,000 |  | 5 | 400 | 2,175* |  | 16 | 2,227 | 3,290* |  | 17 | 715* | 38,829* |
|  |  | Untreated | 2 | 800 | 3,950 |  | 5 | 6,550* | 33 |  | 16 | 1,188 | 1,816 |  | 17 | 332 | 7,250 |
|  |  |  |  |  |  |  |  |  |  |  |  |  |  |  |  |  |  |
| SS | Bogandé^e^ | Treated | - | - | - |  | - | - | - |  | 14 | 328 | 3,995* |  | 23 | 1,217 | 9,257 |
|  |  | Untreated | - | - | - |  | - | - | - |  | 14 | 235 | 1,448 |  | 23 | 1,175 | 7,446 |

^a^ NGS: Northern Guinea Savannah; SGS: Southern Guinea Savannah; SS: Sahel Savannah

^b^ CFU: Colony forming units

^c^ Values with an asterisk (*) were significantly higher than their corresponding paired sample by Student’s *t*-test (α=0.05)

^d^ Experiments on groundnut fields in Dandé in 2012 were not conducted

^e^ Experiments on maize fields of Bogandé were not conducted in either year
